# Radiographic calibration for pubic diastasis assessment in bladder exstrophy-epispadias complex: a phantom study

**DOI:** 10.1007/s00247-024-05972-y

**Published:** 2024-06-27

**Authors:** S. J. Back, D. A. Weiss, B. Marshall, E. Akbari, M. Mackey, E. Hinton, B. D. Horn, M. Kidd, M. L. Francavilla

**Affiliations:** 1 Department of Radiology, Children’s Hospital of Philadelphia, Philadelphia, PA USA; 2 Division of Urology, Children’s Hospital of Philadelphia, Philadelphia, PA USA; 3 Division of Orthopedic Surgery, Children’s Hospital of Philadelphia, Philadelphia, PA USA; 4 Perelman School of Medicine, University of Pennsylvania, Philadelphia, PA USA; 5 Department of Radiology, University of South Alabama, Whiddon College of Medicine, Mobile, AL USA; 6 Centre for Statistical Consultation, University of Stellenbosch, Stellenbosch, South Africa

**Keywords:** Bladder exstrophy, Calibration, Pubic symphysis diastasis, Radiography

## Abstract

**Background:**

The assessment of pubic diastasis is important for the surgical planning of patients with bladder exstrophy-epispadias complex. Understanding how the diastasis changes during surgical follow-up may help predict patient morbidity. Radiography can follow diastasis but may be affected by patient and technical imaging factors including body size, imaging protocol, and equipment. Using imaging calibration and anatomic ratios may mitigate differences due to these aspects.

**Objective:**

Use imaging phantoms to assess the effect of radiographic calibration on measurements of pubic diastasis and an internal anatomic ratio as a child grows.

**Materials and methods:**

Radiographic images were obtained of three different sizes of computed tomography phantoms (older child, child, and infant) using three imaging techniques that include the osseous pelvis in children. All phantoms were imaged with abdomen and pelvis techniques. The infant phantom was additionally imaged using a thoracoabdominal technique. These exposures were all repeated with systems from three manufacturers. Linear measurements were made between radiographic markers placed to simulate pubic diastasis and sacral width. A ratio was also created between these distances. Measurements with and without image calibration were made by two pediatric radiologists using rulers placed at the time of image acquisition.

**Results:**

There was excellent interrater agreement for measurements, ICC >0.99. Anterior distances were more affected by magnification than posterior ones with a significant difference between uncalibrated versus calibrated anterior distances (*p*=0.04) and not for posterior ones (*p*=0.65). There was no difference between radiographic equipment manufacturers without or with calibration (*p* values 0.66 to 0.99). There was a significant difference in simulated pubic distance between thoracoabdominal and abdomen (*p*=0.04) as well as pelvic (*p*=0.04) techniques which resolved with calibration, each *p*=0.6. The ratio between the simulated pubic diastasis and sacral width differed by phantom size (all *p*<0.01) and imaging technique (*p* values 0.01 to 0.03) with or without calibration. However, the numerical differences may not be clinically significant.

**Conclusion:**

Image calibration results in more uniform measurements that are more accurate than uncalibrated ones across patient size, imaging techniques, and equipment. Image calibration is necessary for accurate measurement of inter-pubic distances on all projection imaging. Small differences in the pelvic ratio likely are not clinically significant, but until there is a better understanding, image calibration may be prudent.

**Graphical abstract:**

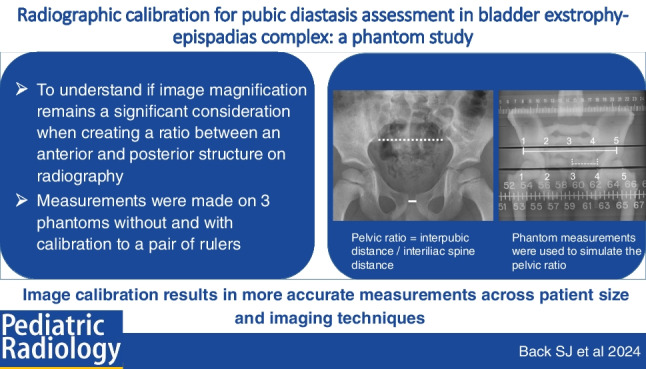

## Introduction

Bladder exstrophy-epispadias complex (EEC) encompasses a spectrum of congenital anomalies that involve the genitourinary and musculoskeletal systems. Patients with EEC have multiple pelvic bone abnormalities including pubic diastasis [1]. Those with isolated epispadias have less pubic diastasis than those with cloacal exstrophy, suggesting that the soft tissue and musculoskeletal severity may be related.

Surgical goals include closure of the midline defects, achievement of urinary continence with preservation of renal function, and an acceptable appearance of the genitalia. The operative success of soft tissue reconstruction is improved if the pelvic ring is reconstructed with or without osteotomies, and tension can be reduced on the midline repair [2]. Since pubic diastasis has been associated with pelvic floor musculature deformities, some postulate that the degree of diastasis can predict short-term complications and influence long-term outcomes [3]. Therefore, measurement of the pubic diastasis is important for preoperative planning. Pubic diastasis has also been observed to increase, both shortly after repair and progressively over time. This dynamic change has led to consideration if early changes in diastasis are related to risk of surgical complications and if late changes predict challenges with pelvic floor support for continence and pelvic organ prolapse. Pelvic organ prolapse, for example, has been associated with the degree of pubic diastasis, but not necessarily osteotomy. Re-doing osteotomies has been presented as a treatment and therefore having accurate measurements of diastasis would be valuable [4, 5]. Thus, there is speculation as to whether measuring changes in diastasis over time can inform the course of patient care.

Abdominal and pelvic radiographs of patients with EEC are routinely obtained from the neonatal period to adulthood. However, these can be obtained with varied techniques and following pubic diastasis over time is confounded by expected patient growth coupled with the inherent magnification of projection imaging.

**Fig. 1:**
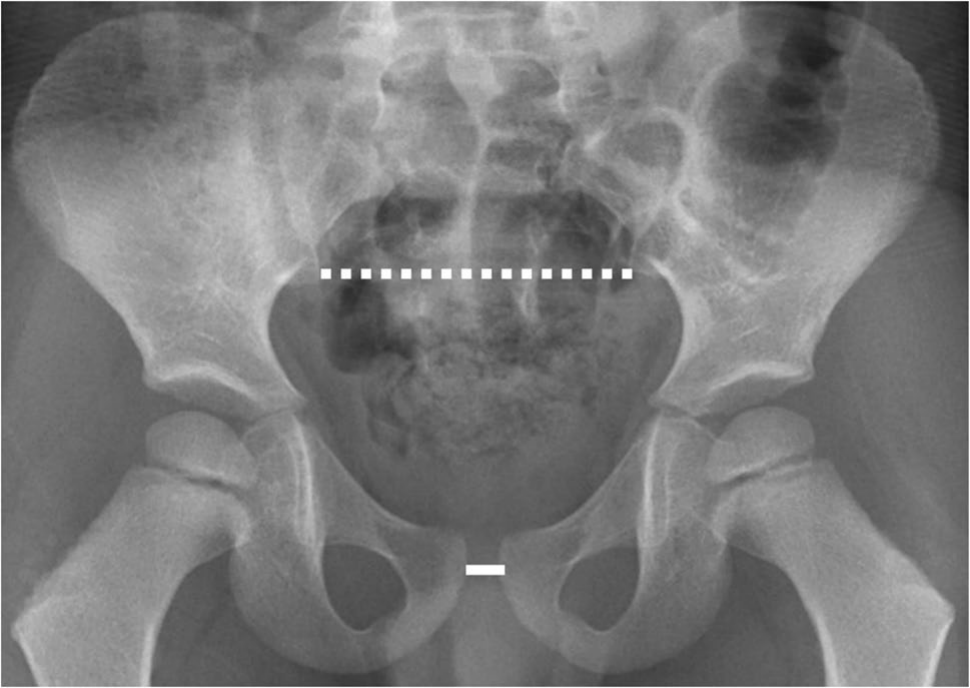
Pelvic ratio measurement example on a clinical radiograph. Normal anterior-posterior pelvic radiograph of a 4-year-old boy with knee pain. Dashed line indicates the distance between the posterior inferior iliac spines (the sacral width at a prescribed, recognizable location) and solid line indicates the inter-pubic distance. The pelvic ratio is the inter-pubic distance divided by the sacral width

From CT studies, the sacral width of patients with EEC did not differ from controls [1, 6]. The pelvic ratio, which is a ratio of the transverse pubic distance divided by the transverse distance (width) of the sacrum, has been made on radiograph to standardize measurement of pubic diastasis to enable comparison between individuals and to avoid variation related to patient size and radiographic technique [7–10] (Fig. 1). However, in these reports, they did not consider or correct for projection magnification. Among urology groups researching ECC, some have suggested that image calibration is necessary to correct for magnification that otherwise makes the measurements and ratios inaccurate. This phantom study was designed to understand if image magnification remains a significant consideration when creating a ratio between an anterior and posterior structure on radiography.

## Materials and methods

**Fig. 2:**
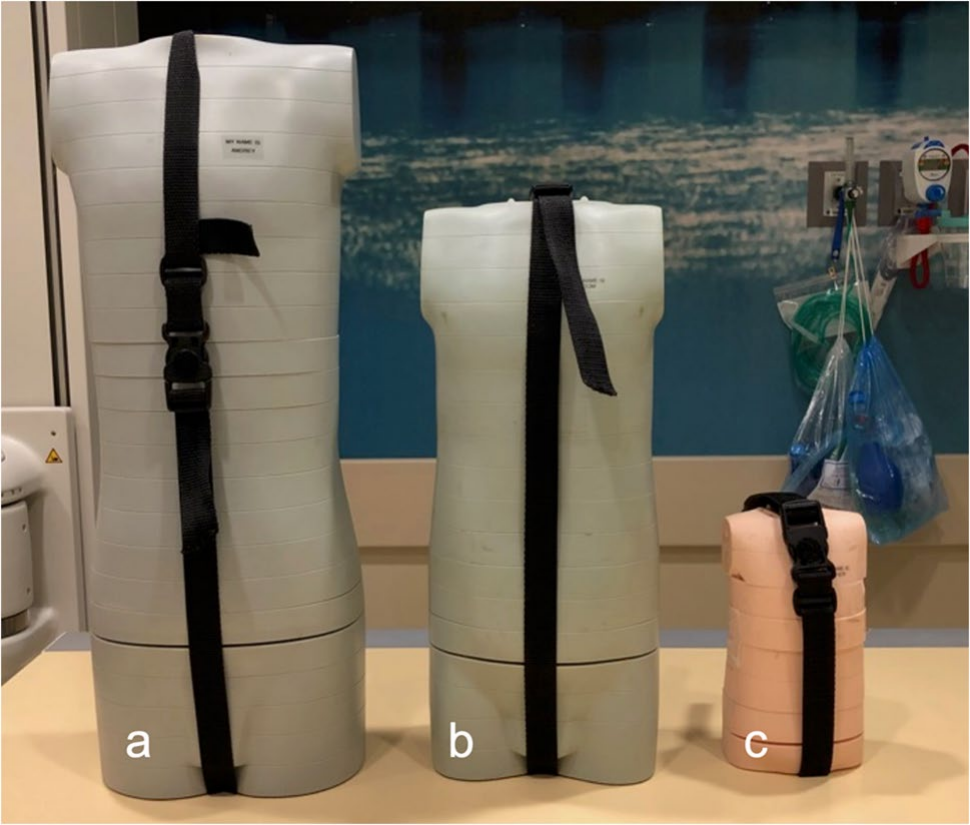
CT phantoms used to obtain radiographs. Photograph of the older child (**a**), child (**b**), and infant (**c**) CT phantoms. The radiographic (BB) markers were placed in between the phantom “slices” as seen in Fig. 3

**Fig. 3:**
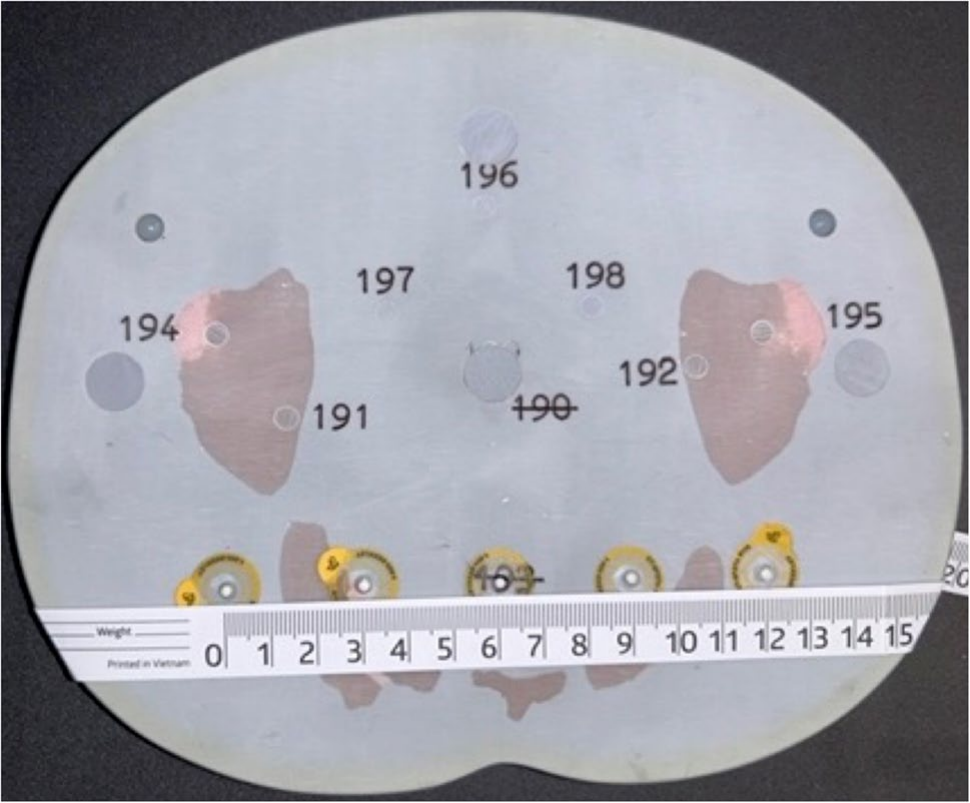
Example of radiographic marker placement on a CT phantom. Photograph of the posterior marker placement on a “slice” of the “older child” phantom in the approximate plane of the posterior inferior iliac spine. The markers (central white circle, within the yellow circles) were placed at 30-mm intervals. The longest distance between markers, 120 mm, is referred to as the “long” measure. The whole numbers on the measuring tape denote centimeter measurements

**Fig. 4:**
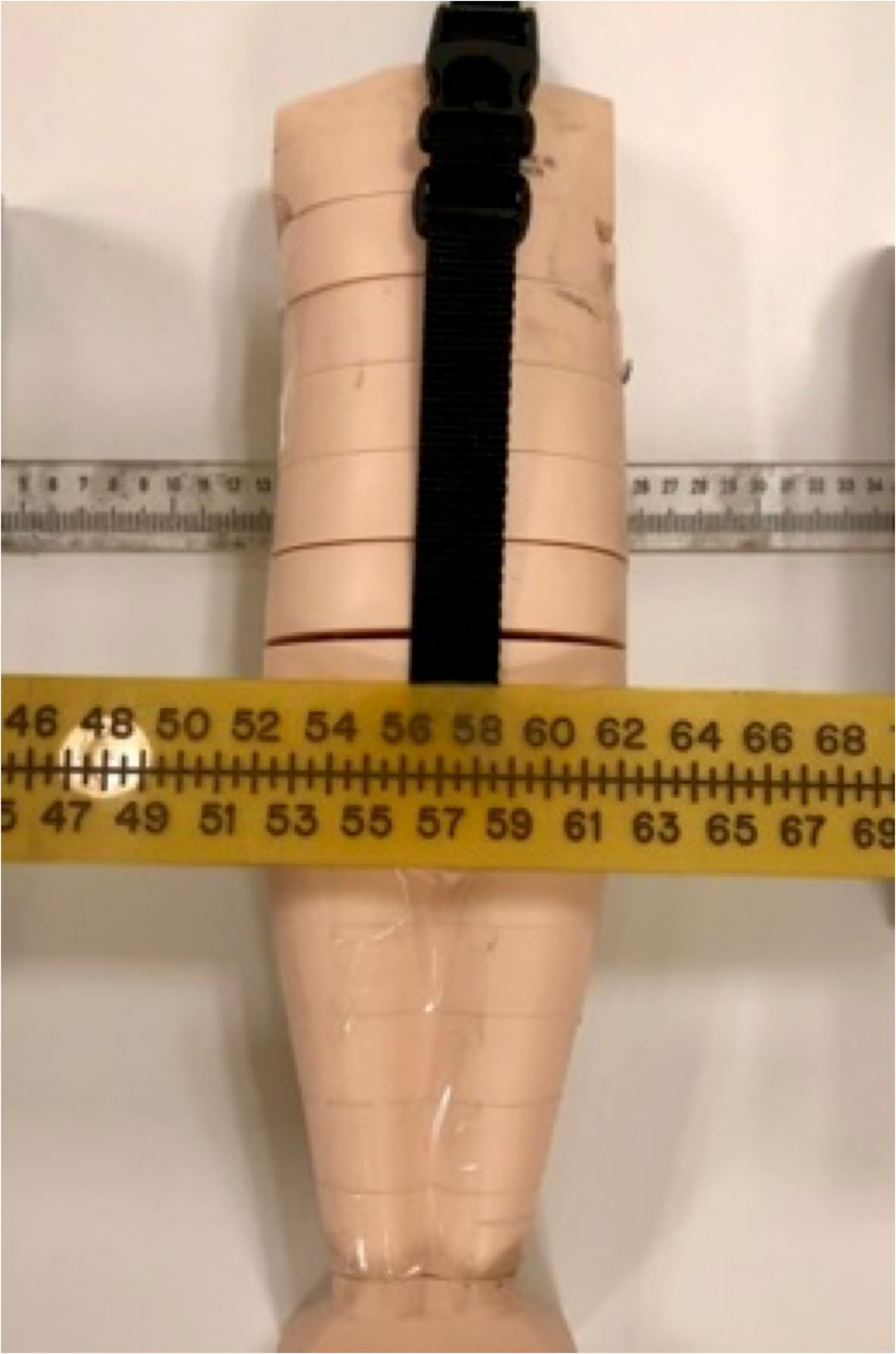
Phantom positioning for radiography. Photograph of the infant-size CT phantom with posterior (clear translucent, cephalad) ruler placed on the tabletop and anterior (yellow opaque, caudad) ruler resting on the upper thigh of the phantom. The radiographic (BB) markers were placed in between the “slices” of the phantom just above the anterior ruler and below the posterior ruler which resulted in a small air gap between the phantom slices

As this was a phantom study, institutional review board approval was not required. Three to five radiopaque imaging markers (CT-Spot® 2.3 mm non-metallic adhesive markers, Beekley Medical, Bristol, CT) were placed in a line at 30-mm intervals within infant-, child (approximately 5 years of age)-, and older child (approximately 10 years of age)-sized CT phantoms (CIRS Inc, Norfolk, Virginia) (Fig. 2). The markers were placed at the approximate depth of the pubic symphysis (anterior) and posterior inferior iliac spines (posterior) as determined based on the phantom construction (Fig. 3). The shortest distance between two markers in a plane is referred to as the “short” measure and is 30 mm for all phantoms. The longest distance between the two markers in a plane is referred to as the “long” measure. In the child and older child phantoms, the long distance was 120 mm (between markers 1 and 5), but the infant phantom could only accommodate three markers, so the long distance was 60 mm. Rulers were placed on the tabletop (posterior ruler) and atop the phantom at the level of the thighs (anterior ruler) (Fig. 4).

The phantoms were radiographed using radiographic techniques performed in clinical practice per the child’s size, including the pelvis. All phantoms were imaged with abdomen and pelvis techniques. The infant phantom was additionally imaged using a thoracoabdominal (“babygram”) technique. These exposures were all repeated with systems from three manufacturers (GE HealthCare, Chicago, IL; Philips Healthcare, Cambridge, MA; Siemens Healthineers AG, Forchheim Germany). A source to image distance of 40 inches was used for all techniques and each phantom on all equipment. Two units (Philips, Siemens) were installed in the radiology department; therefore, there was a 2-inch gap between the phantom and the image detector. A third, portable unit (GE) was brought into the radiography suite. The phantom was placed directly on the detector for this unit.

**Fig. 5:**
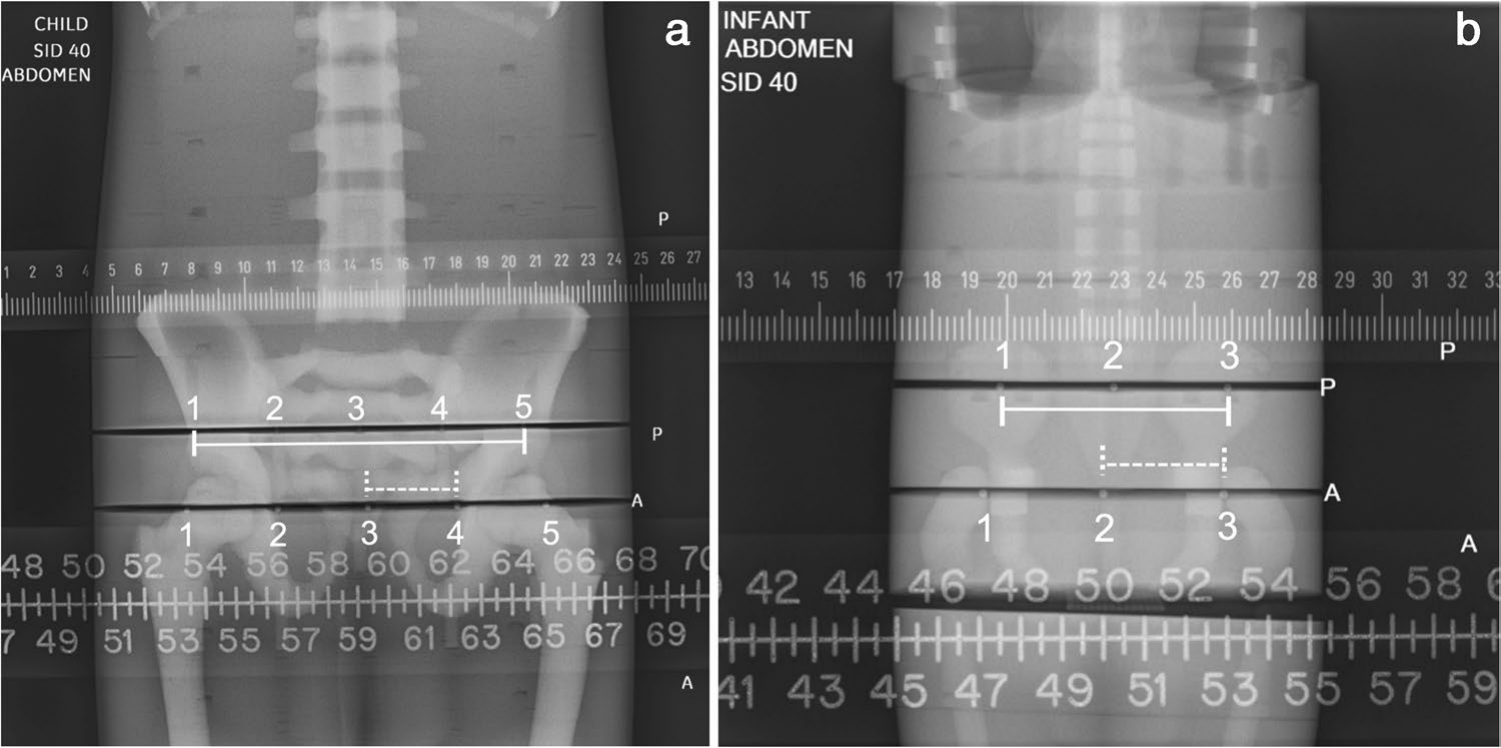
Examples of anterior-posterior abdominal radiographs of two phantoms. Older child (**a**) and infant (**b**). Image annotations were placed by the performing technologist. The phantom size (infant, child, or older child) and radiographic technique (thoracoabdominal, abdomen, pelvis) were labeled in the left upper aspect of each image. The anterior and posterior radio-opaque markers were numbered, left to right, 1 through 5 for the child and older child (**a**) and 1 through 3 for the infant (**b**). The short measure was made between markers 3 and 4 for both the child and older child (**a**) and between markers 2 and 3 for the infant (**b**) for both the anterior and posterior marker positions. These figures show the short measure along the anterior markers (dashed line). The long measurements were made between markers 1 and 5 for the child and older child phantoms (**a**) and between 1 and 3 for the infant phantom (**b**) for both the anterior and the posterior marker positions In these figures the long measure is demonstrated along the posterior markers (solid line). The ratio of the short anterior measure and the long posterior measure simulated the pelvic ratio. P, posterior ruler and posterior radio-opaque markers; A, anterior ruler and anterior radio-opaque markers

Image annotations were placed by the performing technologist (Fig. 5). The phantom size and radiographic technique were labeled in the left upper aspect of each image. A “P” was placed next to the posterior ruler and line of posterior radio-opaque markers, and an “A” was placed next to the anterior ruler and line of anterior radio-opaque markers. The anterior and posterior markers were numbered, left to right, 1 through 3 for the infant and 1 through 5 for the child and older child (Fig. 5). The short measure was made between markers 2 and 3 for the infant and between markers 3 and 4 for both the child and older child. The long measurements were made between markers 1 and 3 for the infant phantom and between 1 and 5 for the child and older child phantoms (Fig. 5).

Two board-certified pediatric radiologists with 6 and 8 years of subspecialty experience evaluated the radiographs in the clinical picture archiving and communications system (PACS) (IntelliSpace PACS, Koninklijke Philips N.V., Amsterdam, Netherlands). Each recorded the anterior and posterior short and long measures without calibrating the image. Subsequently, the image was calibrated using the built-in PACS calibration tool according to the posterior ruler, and the posterior short and long distances were recorded. The calibration process was repeated using the anterior ruler, and the anterior short and long distances were again recorded. In all subsequent calculations, calibration refers to calibration of the posterior measurements to the posterior ruler and calibration of the anterior measurements to the anterior ruler.

The inter-pubic distance is shorter than the distance between the posterior inferior iliac spine distance. Therefore, the pelvic ratio was simulated by assessing the anterior short distance (inter-pubic or IP distance) divided by the posterior long distance (interiliac spine or IS distance). The pelvic ratio (IP/IS) was defined by the following equations: 

$${\mathrm{Pelvic}\;\mathrm{ratio}}_{\mathrm{uncalibrated}}=\frac{\mathrm{short}\;{\mathrm{measure}}_{\mathrm{uncalibrated}}}{\mathrm{long}\;{\mathrm{measure}}_{\mathrm{uncalibrated}}}\;\mathrm{and}\;{\mathrm{Pelvic}\;\mathrm{ratio}}_{\mathrm{calibrated}}=\frac{\mathrm{short}\;{\mathrm{measure}}_{\mathrm{calibrated}}}{\mathrm{long}\;{\mathrm{measure}}_{\mathrm{calibrated}}}$$

Because the maximum long measurement in the infant phantom was half of that attainable in the child and older child phantoms, the posterior long measure of the infant was doubled when creating the pelvic ratio of the infant phantom for appropriate comparison with the other phantom sizes. Additionally, to compare the phantom (body) size and radiographic technique (thoracoabdominal, abdominal and pelvic radiographs), only the short measures were considered.

Interrater agreement was assessed with intraclass correlation coefficients (ICC). Differences between various measures were assessed using a linear mixed-effects model ANOVA with the phantoms as random effect, and other effects like calibration, length, manufacturer, and image technique as fixed effects. Normal probability plots were inspected for normality assumptions and judged to be acceptable. Post hoc analyses were done using Fisher least significant difference (LSD) testing. ICCs were calculated using the R package “irr”. Mixed models were conducted using the “lme4” package in R. Graphs were constructed with Statistica 14 (Cloud Software Group, Fort Lauderdale FL). A *p*-value <0.05 was considered statistically significant.

## Results

### Interrater agreement

Across all uncalibrated and calibrated measurements, including all phantoms and radiographic techniques, made by the two observers, agreement was excellent (ICC >0.99, SEM=0.246, confidence interval=(0.999918, 0.999973)).

### Calibration

All uncalibrated measurements, both long and short, were always longer than the calibrated measures.

### Short versus long measures

The difference between uncalibrated short (mean ± SD: 33.7 mm ± 2.13) versus calibrated short measures (mean ± SD: 30.2 mm ± 0.92) was not significant (*p*=0.19), while the difference between uncalibrated long (mean ± SD: 106.5 mm ± 36.7) versus calibrated long (mean ± SD: 94.6 mm ± 30.7) measures was significant (*p* <0.01)

### Anterior versus posterior position

Differences between uncalibrated (mean ± SD: 73 mm ± 46.8) and calibrated (mean ± SD: 60.4 mm ± 37.2) anterior measures were significant (*p*=0.04), while differences between uncalibrated (mean ± SD: 67.2 mm ± 42.6) and calibrated (mean ± SD: 64.4 mm ± 40.7) posterior measurements were not (*p*=0.65).

### Radiographic equipment

Without calibration, the measurements from different radiographic vendors were not significantly different (mean ± standard deviation): GE 67.3 mm ± 43.0; Philips 72.3 mm ± 46.5; Siemens 70.8 mm ± 45.3(*p*=0.66 to 0.89). However, calibration made the values nearly identical (mean ± standard deviation): GE 62.6 mm ± 39.2; Philips 62.3 mm ± 39.1; Siemens 62.4 mm ± 39.1 (*p*=0.98 to 0.99).

### Phantom size

There was a significant difference between the short measure of each phantom regardless of calibration (all *p*≤0.01) (Table 1).

**Table 1:** Comparison of short measurements, uncalibrated and calibrated, on each phantom (infant, child, and older child). *SD* standard deviation

Phantom size	Uncalibrated Mean (mm) ± SD	Calibrated Mean (mm) ±SD	*P*-value
Infant	32.6 ± 1.34	29.9 ± 0.52	<0.01
Child	33.9 ± 1.81	30.2 ± 1.02	<0.01
Older child	35.3 ± 2.45	30.6 ± 1.2	<0.01

### Radiographic technique

The uncalibrated short measures of the thoracoabdominal radiograph significantly differed from the uncalibrated short measures of the abdomen (*p* = 0.04) and pelvis (*p*=0.04) techniques. However, with calibration, there was no significant difference between thoracoabdominal and abdomen (*p*=0.6) or between thoracoabdominal and pelvis (*p*=0.6). There was no significant difference between the abdomen and pelvis techniques, uncalibrated (*p*=0.96) or calibrated (0.98) (Table 2).

**Table 2:** Comparison of short measurements, uncalibrated and calibrated, on each imaging technique: thoracoabdominal (babygram), abdomen and pelvis. *SD* standard deviation

Imaging technique	Uncalibrated mean (mm) ± SD	Calibrated mean (mm) ± SD	*P*-value
Thoracoabdominal	32.6 ± 1.40	29.9 ± 0.52	<0.01
Abdomen	33.9 ± 2.24	30.2 ± 0.92	<0.01
Pelvis	33.9 ± 2.16	30.2 ± 1.03	<0.01

### Pelvic ratio

The pelvic ratio (IP/IS), a unitless measure, was significantly different based on phantom size, both uncalibrated and calibrated (all *p*<0.01). The absolute difference between the uncalibrated and calibrated pelvic ratio means for each phantom ranged from 0.023 to 0.048 (Table 3).

**Table 3:** Pelvic ratio based on phantom size and calibration. Comparison of uncalibrated and calibrated inter-pubic to sacral width ratio (pelvic ratio) based on phantom size (infant, child, or older child). The pelvic ratio is a unitless measure. *SD* standard deviation

Phantom size	Uncalibrated mean ± SD	Calibrated mean ± SD	*P*-value
Infant	0.266 ± 0.0016	0.243 ± 0.0016	<0.01
Child	0.273 ± 0.0019	0.235 ± 0.0019	<0.01
Older child	0.280 ± 0.0014	0.232 ± 0.0019	<0.01

The pelvic ratio was significantly different for the thoracoabdominal technique compared to the abdomen when uncalibrated (*p*=0.01) and calibrated (*p*=0.03). Similarly, the ratio was significantly different between the thoracoabdominal and pelvic techniques when uncalibrated (*p*=0.02) and calibrated (*p*=0.02). However, the pelvic ratio of the abdomen and pelvis techniques were not significantly different without (*p*=0.68) or with (*p*=0.72) calibration. The absolute difference between the uncalibrated and calibrated pelvic ratio means for each imaging technique ranged from 0.024 to 0.037 (Table 4).

**Table 4:** Pelvic ratio based on imaging technique and calibration. Comparison of uncalibrated and calibrated inter-pubic to sacral width ratio (pelvic ratio) based on radiographic technique: thoracoabdominal (babygram), abdomen, or pelvis. The pelvic ratio is a unitless measure. *SD* standard deviation

Imaging technique	Uncalibrated mean ± SD	Calibrated mean ± SD	*P*-value
Thoracoabdominal	0.267 ± 0.0021	0.243 ± 0.0013	<0.01
Abdomen	0.274 ± 0.0062	0.237 ± 0.0051	<0.01
Pelvis	0.273 ± 0.0061	0.236 ± 0.0048	<0.01

## Discussion

Pubic bone diastasis in patients with ECC correlates with significant differences in the pelvic floor musculature. The osseous relationships change over the course of surgical reconstruction and long-term follow-up [3]. While not previously demonstrated in the literature, to our knowledge, we postulate that a wider or narrower diastasis is associated with more or less severe pelvic floor deformity which may portend the risk of complications and influence both short- and long-term outcomes. As radiography is a mainstay imaging application for patients with ECC, understanding the osseous anatomy and potentially quantifying these relationships prior to and during reconstruction may help further guide treatment. Moreover, pubic diastasis after various osteotomies and traction methods has been associated with outcomes, which led to the use of the degree of diastasis as a measure of how well the osteotomy or traction worked [11, 12]. However, these studies do not describe calibration or an internal ratio to standardize the measurements, and as such, these measurements may not be an accurate depiction of what is occurring with the bones. We designed this phantom study using different exam techniques and body sizes to simulate the radiographic measurement of pubic diastasis; the linear distance between the posterior inferior iliac spines; and the creation of a ratio between these values to understand the differences between uncalibrated and calibrated measurements. To determine if radiographic measurements can be compared to one another over the growth and development of a child, we considered several questions. First, can measurements from different radiographic equipment and techniques (thoracoabdominal image of a neonate, abdomen or dedicated pelvic radiographs) can be compared to one another? In particular, we wanted to know if the portable radiographic units used to obtain images in the neonatal intensive care unit and operating room are comparable to the fixed units used for outpatient imaging follow-up. Second, can calibration mitigate the effects of magnification when making measurements on radiographs? As an anterior structure, the measurement of pubic diastasis is subject to greater magnification than measurements of posterior anatomy. Magnification is increased during growth and development as the distance between the pubis and the radiographic image detector becomes greater with increased pelvic girth. Lastly, can creating a ratio using an internal measurement standardize and account for these variables?

In this phantom study with excellent interrater agreement, we found statistically significant differences in the absolute measures with the use of calibration. Calibrated measurements were shorter than uncalibrated ones and closer to the true distance.

Here, calibration also helped to correct this projection magnification associated with the pubis being an anterior structure with greater object to image distance than for the posterior inferior iliac spines (Fig. 5). The differences between calibrated versus uncalibrated measurements increased as the anteroposterior diameter of the phantom (child) increased with age and were also corrected with calibration, and were as high as 12.5 mm difference in anterior measures. Uncalibrated measurements of the abdomen and pelvic radiographic techniques were similar, whereas there were differences between uncalibrated measures of these techniques compared to the thoracoabdominal technique. However, with calibration, there was no difference between any of these techniques. These observations support the use of calibration when comparing measurements obtained via different radiographic techniques and on differing sized children.

Here, we standardized the source to image distance for the installed and portable radiography units. However, the portable unit has an inherently smaller object to image distance, as the phantom (child) is positioned on top of the detector while the departmental units have an additional 2 inches between the anatomy and the image. Calibration made measurements obtained from different equipment more uniform.

Normative reports of pubic distance were done by assessing abdominal or pelvic radiographs in 888 children aged 0 to 16 years, where no difference was found in pubic distance between genders or radiographic techniques when comparing measures of patients who underwent both studies [13]. Without considering projection magnification, the inter-pubic distance was greatest in infants but remained below 1 cm throughout childhood with a relatively consistent distance from 4 to 16 years of age [13]. McAlister et al. assessed pubic distance to establish a normative range in children in order to recognize diastasis, with a suggested maximal diameter of 0.84 mm using an electronically calibrated measuring tool [14]. While the authors concur that there is no significant difference between male and female children, one group did not account for the effects of image magnification and the other used the electronically calibrated measuring tool of the imaging system. The necessity of calibration depends on the acceptable measurement error for a given application.

Radiography is a common imaging modality in both pre- and postoperative assessment in fields like orthopedics and dentistry. Magnification has been a significant concern in the preoperative measurements made for templating prosthetic size for adult total hip arthroplasty and in the postoperative evaluation of prosthetic subsidence because small distances (2–4 mm) are clinically significant [15]. In a cohort of patients who underwent orthopedic fixation for trauma, there was magnification of the implanted hardware, between 13 and 29% compared with the known size, which was greatest in central anatomy (proximal femur) [16]. In a study of a length standard phantom for dental radiographs, the authors report a deviation in the linear length of −3.37 ± 0.15 mm without calibration and improvement to −0.11 ± 0.10 with image calibration [17].

One approach to standardize measurements for comparison between patients has been to create a ratio between the desired measurement and a common denominator. This was applied to the assessment of ulnar variance in a group of adult patients with distal radial fractures. Ulnar variance was divided by the capitate height which served as an internal control for images that were uncalibrated or unscaled [18]. However, if magnification and variables that may affect magnification (body size, exam technique, radiographic equipment) have significant effect on the ratio, then a standardized and calibrated approach is necessary to assess such measurements for use in clinical care.

There are limitations to our study. As a phantom design, measurements of clinical radiographs will be needed to confirm these observations. When placing a standard or calibration ruler, it is ideal for this object to be at the same level as the anatomy that is being measured. While we have positioned two separate rulers in this study, our positioning does not account for soft tissue thickness, which would limit the ruler position relative to the bone anatomy. Many orthopedic practices utilize a height-adjustable spherical fiducial marker for calibration of pelvis and leg-length radiographs. Unlike a ruler placed atop a patients’ thighs, such a device is not subject to angular deflection and can be placed at the palpated level of the pubic symphysis [19]. We utilized radiographic vendors in our institution and while we think these principles will hold for other vendors, we cannot be certain.

We have confirmed that anterior structures have greater magnification on projection imaging than posterior structures. Based on this magnification difference, the outcome of the pelvic ratios without and with calibration are expected. With the increased numerator on the uncalibrated images, the ratio should be larger when measurements are uncalibrated versus calibrated. However, the pelvic ratios decreased from approximately 0.26 to 0.27 on uncalibrated images to approximately 0.23 to 0.24 when calibrated, a difference of only 0.04. The role of the pelvic ratio as an imaging marker for surgical outcomes is an area of active research. This small numerical difference in the ratio, while statistically significant in this study, may not be clinically relevant. However, if distances are measured on clinical radiographs, we believe it is best to obtain the most accurate measurements, which require calibration. This may be particularly important for research comparing outcomes from different surgical groups with potentially varied imaging techniques and for groups comparing measured pubic diastasis rather than pelvic ratios.

## Conclusions

Image calibration results in more uniform measurements that are more accurate than uncalibrated ones across patient size, imaging techniques, and equipment. Image calibration is necessary for accurate measurement of inter-pubic distances on all projection imaging. Small differences in the pelvic ratio likely are not clinically significant, but until there is a better understanding, image calibration may be prudent.

## Data Availability

Data sets generated during the current study are available from the corresponding author on reasonable request.
